# Over-Expression of PDGFR-β Promotes PDGF-Induced Proliferation, Migration, and Angiogenesis of EPCs through PI3K/Akt Signaling Pathway

**DOI:** 10.1371/journal.pone.0030503

**Published:** 2012-02-15

**Authors:** Hang Wang, Yangguang Yin, Wei Li, Xiaohui Zhao, Yang Yu, Jinkun Zhu, Zhexue Qin, Qiang Wang, Kui Wang, Wei Lu, Jie Liu, Lan Huang

**Affiliations:** 1 Institute of Cardiovascular Science Xinqiao Hospital, Third Military Medical University, Shapingba District, Chongqing, People's Republic of China; 2 Emergency Department, Xinqiao Hospital, Third Military Medical University, Shapingba District, Chongqing, People's Republic of China; Florida International University, United States of America

## Abstract

The proliferation, migration, and angiogenesis of endothelial progenitor cells (EPCs) play critical roles in postnatal neovascularization and re-endothelialization following vascular injury. Here we evaluated whether the over-expression of platelet-derived growth factor receptor-β (PDGFR-β) can enhance the PDGF-BB-stimulated biological functions of EPCs through the PDGFR-β/phosphoinositide 3-kinase (PI3K)/Akt signaling pathway. We first confirmed the expression of endogenous PDGFR-β and its plasma membrane localization in spleen-derived EPCs. We then demonstrated that the PDGFR-β over-expression in EPCs enhanced the PDGF-BB-induced proliferation, migration, and angiogenesis of EPCs. Using AG1295 (a PDGFR kinase inhibitor), LY294002 (a PI3K inhibitor), and sc-221226 (an Akt inhibitor), we further showed that the PI3K/Akt signaling pathway participates in the PDGF-BB-induced proliferation, migration, and angiogenesis of EPCs. In addition, the PI3K/Akt signaling pathway is required for PDGFR-β over-expression to enhance these PDGF-BB-induced phenotypes.

## Introduction

Endothelial injury is a principal factor that contributes to atherosclerosis and restenosis after percutaneous coronary intervention [1], [2]. Endothelial repair and regeneration is therefore a crucial step in treating vascular diseases and a potential target for the therapy. However, advances in therapeutics are limited due to the insufficient de novo differentiation of pre-existing mature endothelial cells (ECs) [3]. Recently, many basic and clinical studies have indicated that the bone marrow-derived endothelial progenitor cells (EPCs) can enter the circulation, differentiate into ECs, and play important roles in postnatal neovascularization and regeneration of the endothelial monolayer [3], [4], [5], [6], [7]. Indeed, EPCs play a fundamental role in neoangiogenesis and re-endothelialization after vascular injury as well as during tumor angiogenesis [8], [9], [10], [11], [12]. Consequently, controls on the number and functional activity of EPCs have become an area of intense investigation.

Platelet-derived growth factor (PDGF) was initially identified in the serum and platelets as a major mitogen for smooth muscle cells and fibroblasts in vitro [13], [14]. PDGF comprises four polypeptides A, B, C, and D, which assemble into disulfide-linked homodimers or heterodimers (PDGF-AA, -BB, -CC, -DD, or PDGF-AB). These five PDGF dimers bind to two PDGF receptors (PDGFR)-α and –β with different affinities [15]. PDGFRs dimerize upon PDGF binding and PDGF-BB is the only PDGF dimer that binds with high affinity to all three PDGFR isoforms: PDGFR-αα, PDGFR-ββ, and PDGFR-αβ. Previous studies have indicated that the interaction between PDGF-BB and PDGFR-β is key for the proliferation and migration of pericytes and the development of a functional vasculature [16], [17]. Moreover, PDGF-BB and PDGFR-β interaction induces the phosphorylation of PDGFR and activates phosphoinositide 3-kinase (PI3K) [18].

The PI3K signaling pathway contributes to a number of cell processes, including cell proliferation, survival, motility, and angiogenesis [19]. Several studies showed that the activation of the PI3K/Akt pathway may play a major role in the statin-induced increase in EPC levels [20], [21], [22]. In addition to PDGF, vascular endothelial growth factor (VEGF) [23], erythropoietin [24], and estrogen [25], [26] can also activate the PI3K/Akt pathway.

To date, the role of PDGFR-β in EPCs still remains largely unknown. In this study, we transfected PDGFR-β into spleen-derived EPCs and found that PDGFR-β over-expression promoted the PDGF-BB-induced proliferation, migration, and angiogenesis of EPCs. We further demonstrated that the PI3K/Akt signaling pathway was critical for both the PDGF-BB-induced activities of EPCs and the promotive effects of PDGFR-β over-expression on the PDGF-BB-induced EPC activities.

## Materials and Methods

### Ethics statement

All procedures were in compliance with the Ethic Committee of Third Military Medical University and the National Institute of Health Guide for the Care and Use of Laboratory Animals.

### EPC culture and characterization

Mouse spleen-derived EPCs were cultured and characterized as previously described [27]. Briefly, spleens were harvested from male C57BL/6 mice (6 to 8 weeks of age, 20 to 25 g of weight, Chongqing, China) and then mechanically minced. Spleen-derived mononuclear cells (MNCs) were isolated using density gradient centrifugation at 2000 g for 20 min. After three times rinses, cells were plated in gelatin-coated cell culture flasks and cultured at 37°C in a 5% CO2 atmosphere with Dulbecco's Modified Eagle Medium: nutrient mixture F-12 (DMEM/F-12) culture medium (Gibco BRL, NY, USA) supplemented with 20% fetal calf serum (FCS, Gibco BRL, NY, USA), 10 ng/mL VEGF (R&D Systems, Minneapolis, MN, USA), 100 U/mL penicillin, and 100 U/mL streptomycin. Starting from four days after culturing, the medium was changed every 3 days. Only attached cells were used for further experiments.

For characterization, differentiating cells were incubated with 2.4 µg/mL acLDL-DiI (Invitrogen, CA, USA) for at 37°C for 4 h and fixed with 4% paraformaldehyde (PFA) for 10 min. After rinsed with phosphate-buffered saline (PBS), cells were incubated with 10 mg/mL FITC-UEA-1 (Sigma-Aldrich, St Louis, MO, USA) for 1 h. Cells that were positive for both acLDL-DiI and UEA-1 were identified as EPCs. Additionally, the phenotypes of EPCs were determined by flow cytometry (FACS). Cells (1×10^6^) were incubated with the following monoclonal antibodies: FITC-conjugated anti-Sca-1 (abCAM, Cambridge, MA, USA), PE-conjugated anti-VEGFR-2 (eBiosciences, San Diego, CA, USA), or their corresponding isotype controls (eBiosciences).

### Semi-quantitative RT-PCR

MNCs were cultured for 4, 7, 14, or 21 days as described above to obtain EPCs. To examine the mRNA expression level of PDGFR-β in transfected cells, EPCs were harvested 72 h post-transfection. Total RNA was extracted with Trizol (Invitrogen) according to the manufacturer's instructions. cDNA was obtained through RT-PCR using a PrimeScript™ RT reagent kit (Takara, Dalian, China), with the total RNA as template, and then amplified. The primers were as follows: PDGFR-β sense: 5′ CCGGCGCTGGCGAGTTAGTTT 3′; PDGFR-β antisense: 5′ ACACCTACTTTTGAGGTCTCTGCAGG 3′; product length: 296 bp. GAPDH sense: 5′ AACTTTGGCATTGTGGAAGGGCTC 3′; GAPDH antisense: 5′ ACCCTGTTGCTGTAGCCGTATTCA 3′; product length: 473 bp. All primers were synthesized by Invitrogen (Shanghai, China).

### Western blot

MNCs were cultured for 4, 7, 14, or 21 days as described above to obtain EPCs. To examine the protein expression level of PDGFR-β in transfected cells, EPCs were harvested 72 h post-transfection. Cells from the transfection groups were used to examine the expression levels of proteins involved in the signaling pathways. The protein content was determined using the Bradford method. Equal amounts of protein were subjected to SDS-PAGE and transferred to PVDF membranes. The membranes were blocked with 5% non-fat milk, probed with anti-PDGFR-β, anti-phospho-PDGFR-β (abCAM), anti-GAPDH, anti-PI3 kinase p85, anti-phospho-PI3 kinase p85, anti-Akt, or anti-phospho-Akt (Cell Signaling Biotechnology, Beverly, MA, USA), and then stained with horseradish peroxidase-coupled secondary antibodies. Protein bands were visualized using enhanced chemiluminescence (Amersham Pharmacia Biotech., UK) and quantified using Quantity One software (Bio-Rad, USA).

### Immunofluorescence

For fluorescence staining, EPCs were fixed with 4% PFA for 15 min. After three times rinses with PBS, cells were permeablized with 0.1% Triton X-100 for 20 min. EPCs were first incubated with an anti-PDGFR-β primary mAb (1∶100) and then with a Cy3-labeled secondary antibody (Beyotime, Shanghai, China). DAPI was used to stain the nuclei of EPCs. Images were taken by laser scanning confocal microscopy (LSCM, Leica).

### Liposome-mediated cell transfection

The plasmid pEGFP-N2-PDGFR-β was kindly provided by Dr. Shangcheng Xu at the Third Military Medical University. After EPCs were cultured for 10 days and reached 60–70% confluence, transfection was performed using a Lipofectamine™ 2000 reagent according to the instruction manual (Invitrogen). The DNA (µg) to Lipofectamine™ 2000 (µL) ratio was 1∶2. Three groups of cells were transfected: (1) control group: no plasmid; (2) pEGFP-N2 group: the plasmid pEGFP-N2; (3) pEGFP-N2-PDGFR-β group: the plasmid pEGFP-N2-PDGFR-β.

Transfected cells can be identified using an in-vector marker, EGFP. In order to evaluate the transfection efficiency, the number of EGFP-positive cells was divided by the total number of EPCs in the same area. Five random view-fields were counted, and the average percentage of transfected cells was calculated.

### Enzyme-Linked Immunosorbent Assay (ELISA) for secreted PDGF-BB

PDGF-BB is a secretory protein, and the concentration of PDGF-BB in the culture medium can be used to estimate the expression level of PDGF-BB in EPCs. To compare the expression level of PDGF-BB between the untransfected and transfected EPCs, we measured the concentration of PDGF-BB in the supernatant of the culture medium using ELISA (R&D Systems) according to the manufacturer's instructions. The supernatant of culture medium were collected at 0, 24, 48 and 72 h after transfection.

### PDGF-BB stimulation and inhibitor pretreatment

To examine the effects of exogenous PDGFR-β transfection and different concentrations of PDGF-BB stimulation on the biological functions of EPCs, the three groups of EPCs from PDGFR-β transfection (the control group, the pEGFP-N2 group, and the pEGFP-N2-PDGFR-β group) were treated with PDGF-BB (Peprotech, USA) of different concentrations (0, 10, 20, 40, 80, or 160 ng/mL) for 24 h. Since results from the control group and the pEGFP-N2 group were similar, only the control group was used for subsequent experiments.

To examine whether the PDGFR-β/PI3K signaling pathway is involved in PDGF-BB-induced biological functions of EPCs, the two groups of EPCs without PDGFR-β transfection (the control group and the pEGFP-N2-PDGFR-β group) were treated as follows: untreated for 24 h; treated with PDGF-BB at maximal effective concentration for 24 h; or pretreated with 20 µM tyrphostin AG1295 (Sigma-Aldrich), 30 µM LY294002 (Cell Signaling Biotechnology), or 30 µM sc-221226 (Santa Cruz Biotechnology, Santa Cruz, CA, USA), respectively, for 1 h before treated with PDGF-BB at maximal effective concentration for 24 h.

### EPC proliferation assay

EPC proliferation was examined by the colorimetric MTS assay (Cell Titer 96 Aqueous, Promega). EPCs were seeded in 96-well plates in quintuplicate at a cell density of 5×10^3^/well and cultured in DMEM/F12 containing 10% FCS. 48 h later, MTS (20 µL/well) was added for another 4 h incubation at 37°C. The optical density (OD) at 490 nm was recorded with a 96-well plate reader.

### EPC migration assay

The migration of EPCs was assayed using a Transwell system (Corning Costar, USA) containing 8 µm polycarbonate filter inserts in 24-well plates. 2×10^5^ cells from the control group, pEGFP-N2 group, or pEGFP-N2-PDGFR-β group without pretreatment or pretreated with AG1295, LY294002, or sc-221226, respectively, for 30 min in 200 µL serum-free DMEM/F12 were placed in the upper chamber. DMEM/F12 containing 10% FCS (500 µL) with or without PDGF-BB was filled in the lower chamber. After 16 h in culture, cells on the bottom of the Transwell membrane were fixed with 4% PFA at 37°C for 20 min and stained with 1% crystal violet at 37°C for 5 min. The number of migrating cells on the bottom of the Transwell in 6 randomly selected high power fields (×200) was counted manually. Results were representative of five independent experiments.

### EPC tube-formation assay

EPC's participation in tube-like formation was evaluated with the In Vitro Angiogenesis Assay Kit (Chemicon, Canada & USA). EPCs were treated as in the migration assay, harvested, resuspended, and reseeded into 96 well plates coated with 50 µL of diluent ECMatrix™ solution at a cell density of 1×10^4^/well. After incubation at 37°C for 6 h, EPC tube-like formation was observed by microscopy, and visual patterns were defined based on the pattern/value association criterion (Table 1). Five randomly selected high power view-fields (×200) per well were assessed and the values averaged.

**Table 1:** Pattern/value association criterion.

Pattern	Value
Individual cells, well separated	0
Cells begin to migrate and align themselves	1
Capillary tubes visible. No sprouting	2
Sprouting of new capillary tubes visible	3
Closed polygons begin to form	4
Complex mesh-like structures develop	5

Cite: In Vitro Angiogenesis Assay Kit instruction.

### Statistical analysis

All data were expressed as mean ± S.D. Statistical analyses were performed with SPSS 13.0 software. Comparisons between multiple groups were tested by Multi-Way ANOVA or One-Way ANOVA. Comparisons between groups were performed using Fisher's LSD test. A probability value of P<0.05 was considered statistically significant.

## Results

### Characterization of spleen-derived EPCs

**Figure 1:**
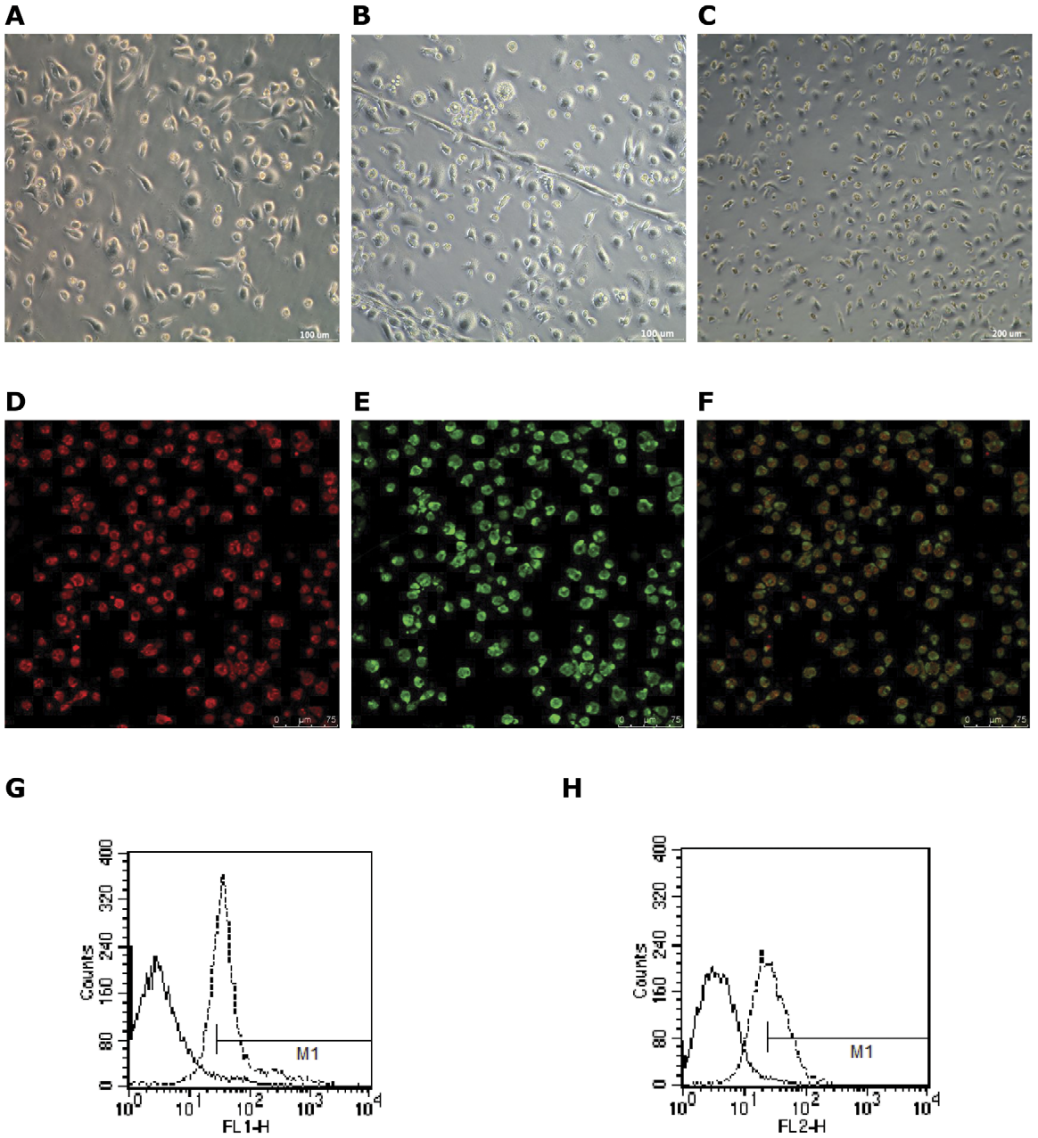
Spleen-derived MNCs differentiate into cells with characteristics of endothelial progenitor cells after cultured in vitro. (**A**) After 4–7days in culture, spleen-derived MNCs exhibited a spindle-shaped, endothelial cell-like morphology. Scale bar = 100 µm. (**B**) After 10 days, cells formed cord-like structures. Scale bar = 100 µm. (**C**) After 20 days, cells became confluent, and looked like cobblestones. Scale bar = 200 µm. (**D,E,F**) Spleen-derived MNCs showed uptake of acetylated LDL (**D**) and UEA-1 binding (**E**) after 4 days in culture. Most adherent cells were LDL and UEA-1 double positive (91.2±1.8%; n = 5; three random fields per well). Scale bar = 75 µm. (**G**) FACS analysis of cultured spleen-derived MNCs for FITC-Sca-1 (a stem/progenitor cell marker), generating a positive rate of 71.7±0.93% (n = 3). (**H**) FACS analysis of cultured spleen-derived MNCs for PE-VEGFR-2 (an endothelial cell marker), generating a positive rate of 52.49±9.27% (n = 3). The left peak in each box denotes corresponding negative isotype control labeling, and the positive gate M1 is shown.

Isolated spleen-derived MNCs exhibited a spindle-shaped morphology after cultured for 4–7 days (Fig. 1A), formed cord-like structures after 10 days (Fig. 1B), and showed like cobblestone like structures after 20 days (Fig. 1C). Most attached cells (91.2±1.8%) were double positive for both acLDL-DiI and UEA-1 and identified as EPCs (Figs. 1D, E, and F). In addition, 71.7±0.93% of these cells expressed mouse stem-cell marker Sca-1 (Fig. 1G), and 52.49±9.27% expressed endothelial cell marker VEGFR-2 (Fig. 1H).

### Endogenous PDGFR-β expression and localization in EPCs

**Figure 2:**
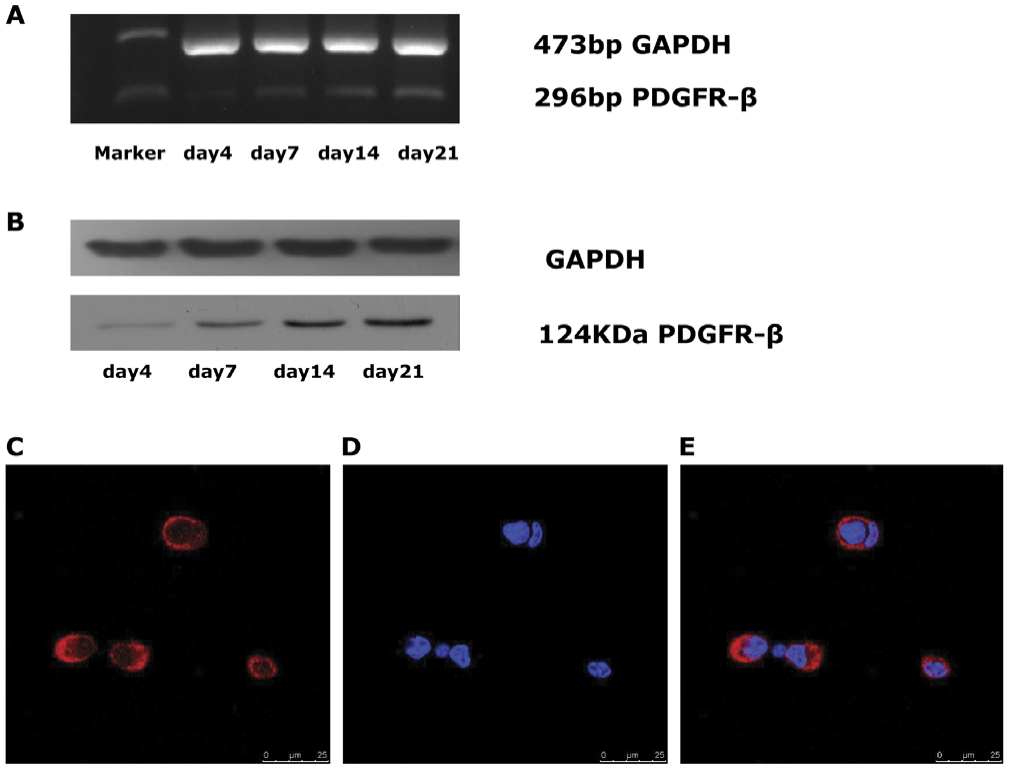
PDGFR-β expression and localization in EPCs. (**A**)RT- PCR analysis of PDGFR-β (296 bp) in EPCs at Day 4, 7, 14, and 21. (**B**) Western blot analysis of PDGFR-β protein (124 kDa) in EPCs at Day 4, 7, 14, and 21. (**C, D, E**) Subcellular localization of PDGFR-β in EPCs. EPCs were stained for PDGFR-β (**C**) and nuclei were stained with DAPI (**D**). PDGFR-β was localized predominantly to the plasma membrane of EPCs. PDGFR-β positive cells in the total cells were 88.6±4.3% (n = 5; three random fields per well).Scale bar = 25 µm.

The expression of PDGFR-β in EPCs progressively increased with differentiation time as shown by semi-quantitative RT-PCR (Fig. 2A) and Western blot (Fig. 2B) using cells from Day 4, 7, 14, and 21. Additionally, the subcellular localization of PDGFR-β in EPCs was investigated by immunofluorescence. PDGFR-β was found to be localized in the plasma membrane of the cells(88.6±4.3%) (Figs. 2C, D, and E). We concluded from these observations that PDGFR-β is expressed in the primary EPCs.

### Over-expression of PDGFR-β in transfected EPCs

**Figure 3:**
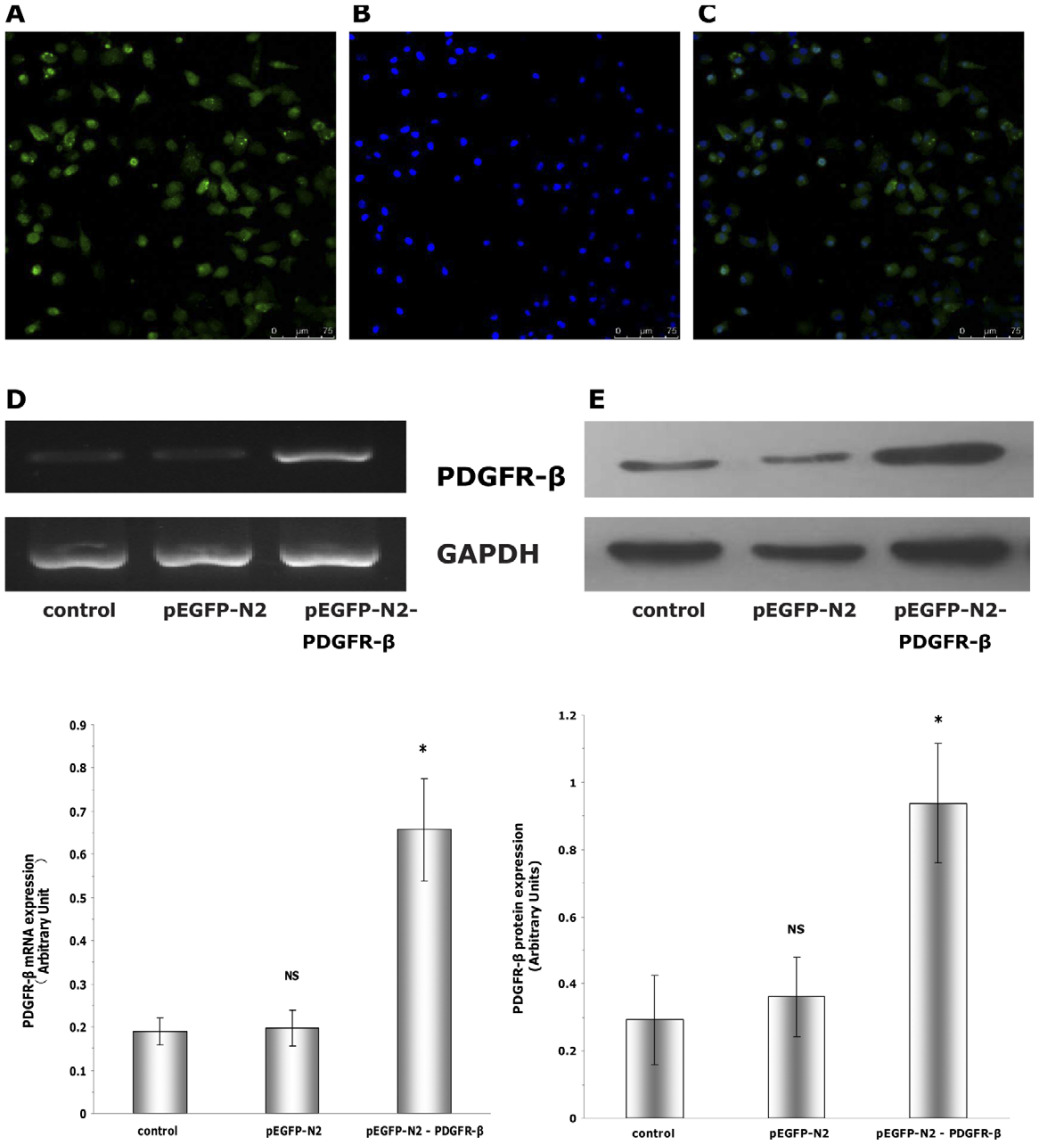
Overexpression of PDGFR-β in transfected EPCs. (**A,B,C**) Transfected cells can be identified using an in-vector marker, EGFP 21 h after transfection, the expression of EGFP was detected by laser scanning confocal microscopy in the pEGFP-N2-PDGFR-β group (**A**), and nuclei were stained with DAPI (**B**). The transfection efficiency (number of green cells/total number of cells) was about 50-60%. Scale bar = 75 µm. (**D,E**) 72 h post-transfection, PDGFR-β mRNA (**D**) and protein levels (**E**) in the pEGFP-N2-PDGFR-β group were significantly higher than in the control group or pEGFP-N2 group.* P<0.01 vs. control or pEGFP-N2; NS, no significant vs. control (n = 3).

EPCs were transfected with either pEGFP-N2 or pEGFP-N2-PDGFR-β. 21 h after transfection, EGFP expression was detected by laser scanning confocal microscopy in both the pEGFP-N2 group and pEGFP-N2-PDGFR-β group, while no EGFP was detected in the control group (data not shown). The transfection efficiency was about 50–60% (Figs. 3A, B, and C). 72 h post-transfection, the level of PDGFR-β in the pEGFP-N2-PDGFR-β group was significantly increased compared to the control group or the pEGFP-N2 group, as shown by semi-quantitative RT-PCR (Fig. 3D) and Western blot (Figs. 3E) (P<0.01).

### Secretion of PDGF-BB by EPCs

**Figure 4:**
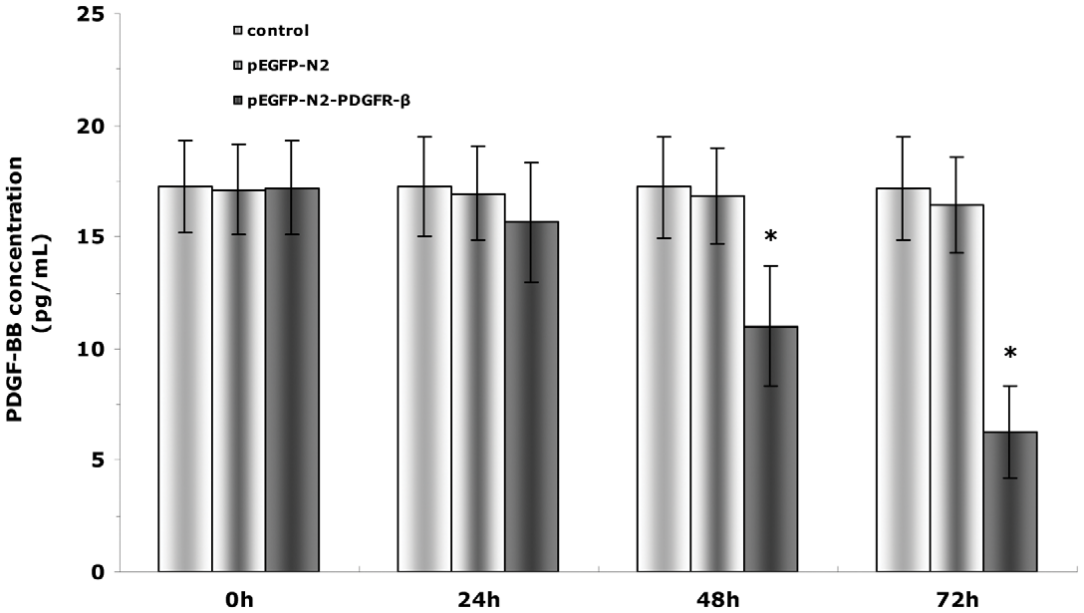
ELISA of PDGF-BB secreted by EPCs in response to PDGFR-β transfection. The concentration of PDGF-BB in the supernatant of culture medium was measured using ELISA. The concentration of PDGF-BB was significantly lower in the pEGFP-N2-PDGFR-β group than in the control or pEGFP-N2 group at 48 h and 72 h post-transfection. * *P*<0.01 vs. control or pEGFP-N2 (n = 6).

In the next step, we sought to determine whether PDGFR-β transfection could change the secretion of PDGF-BB.To this end,we compared the concentration of PDGF-BB in the supernatant of culture medium between the untransfected cells and cells transfected with pEGFP-N2 or pEGFP-N2-PDGFR-β. Our results showed that in cell expressing pEGFP-N2-PDGFR-β, the concentration of soluble PDGF-BB significantly reduced to 11.0±2.7 pg/ml (n = 6) at 48 h post-transfection, and 6.3±2.0 pg/ml (n = 6) at 72 h post-transfection (Fig. 4) (P<0.01).

### Effects of PDGFR-β over-expression on PDGF-BB-induced EPC proliferation

**Figure 5:**
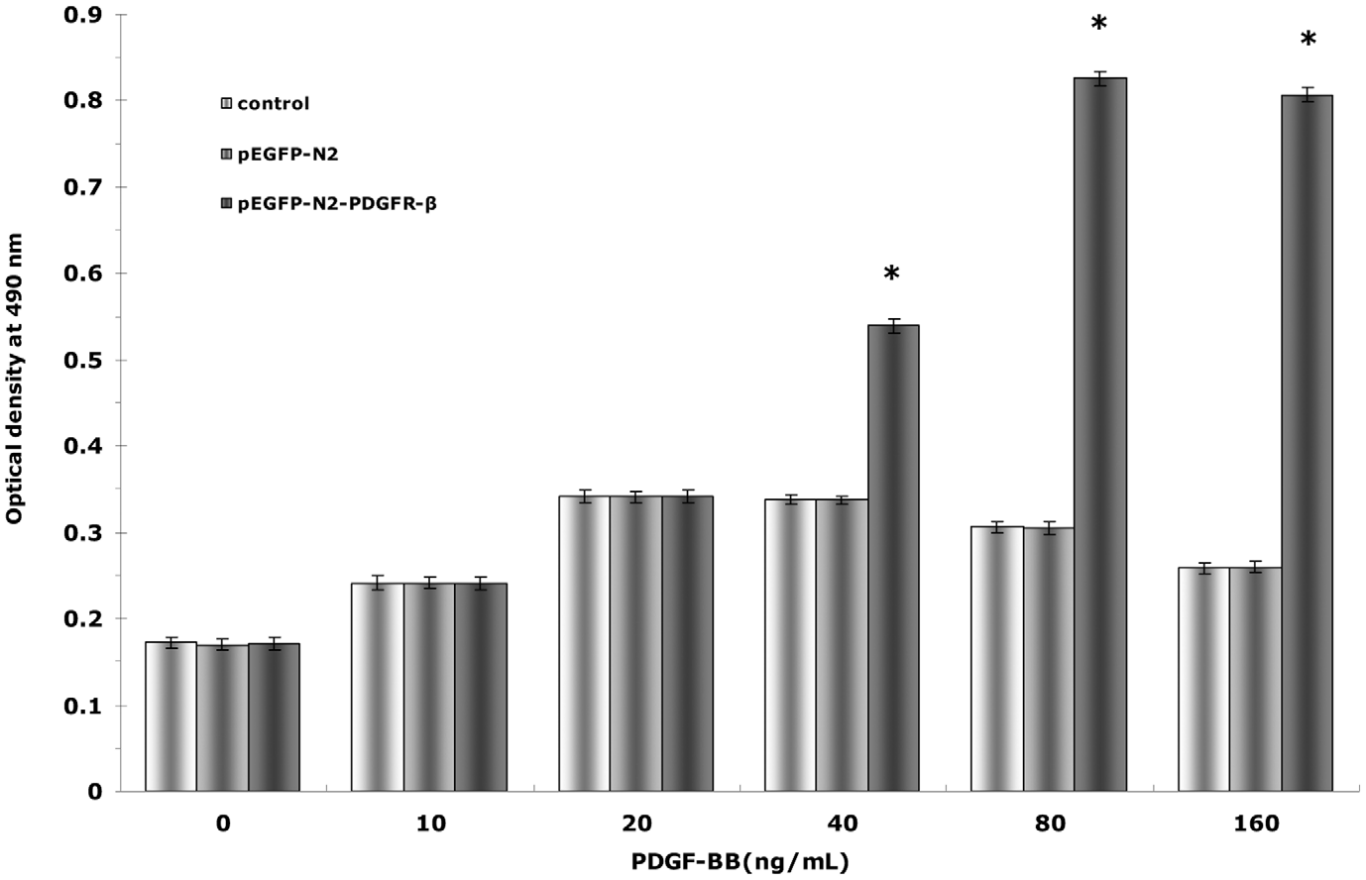
Effects of PDGFR-β overexpression on PDGF-BB-induced EPC proliferation. EPCs (untransfected,or transfected with either pEGFP-N2 or pEGFP-N2-PDGFR-β) were incubated with PDGF-BB of different concentrations.EPC proliferation was examined by the MTS assay. * *P*<0.01 vs. pEGFP-N2-PDGFR-β cells under 0 ng/mL PDGF-BB stimulation.

We next used the MTS assay to examine how the PDGFR-β over-expression affected the EPC proliferation stimulated by recombinant PDGF-BB. The main effects of concentration (F = 220.880, P<0.01) and group (F = 349.826, P<0.01), as well as their interaction (F = 88.717, P<0.01), were all significant. The maximum proliferation induced by recombinant PDGF-BB occurred at 20 ng/mL in both the control and pEGFP-N2 groups and at 80 ng/mL in the pEGFP-N2-PDGFR-β group (Fig. 5). Since no difference was observed between the control and pEGFP-N2 groups, in subsequent proliferation experiments, only the control group and the pEGFP-N2-PDGFR-β group were compared, and the two maximum proliferation concentrations indentified here were used for the two groups to stimulate proliferation, respectively. Interestingly, in the control and pEGFP-N2 groups, EPC proliferation remained unchanged under different concentrations of PDGF-BB (P>0.05) (Fig. 5). In contrast, EPC proliferation increased significantly with increase in PDGF-BB concentration(P<0.05), indicating that PDGF-BB-induced EPC proliferation is enhanced by PDGFR-β over-expression.

### Effects of PDGFR-β over-expression on PDGF-BB-induced EPC migration

**Figure 6:**
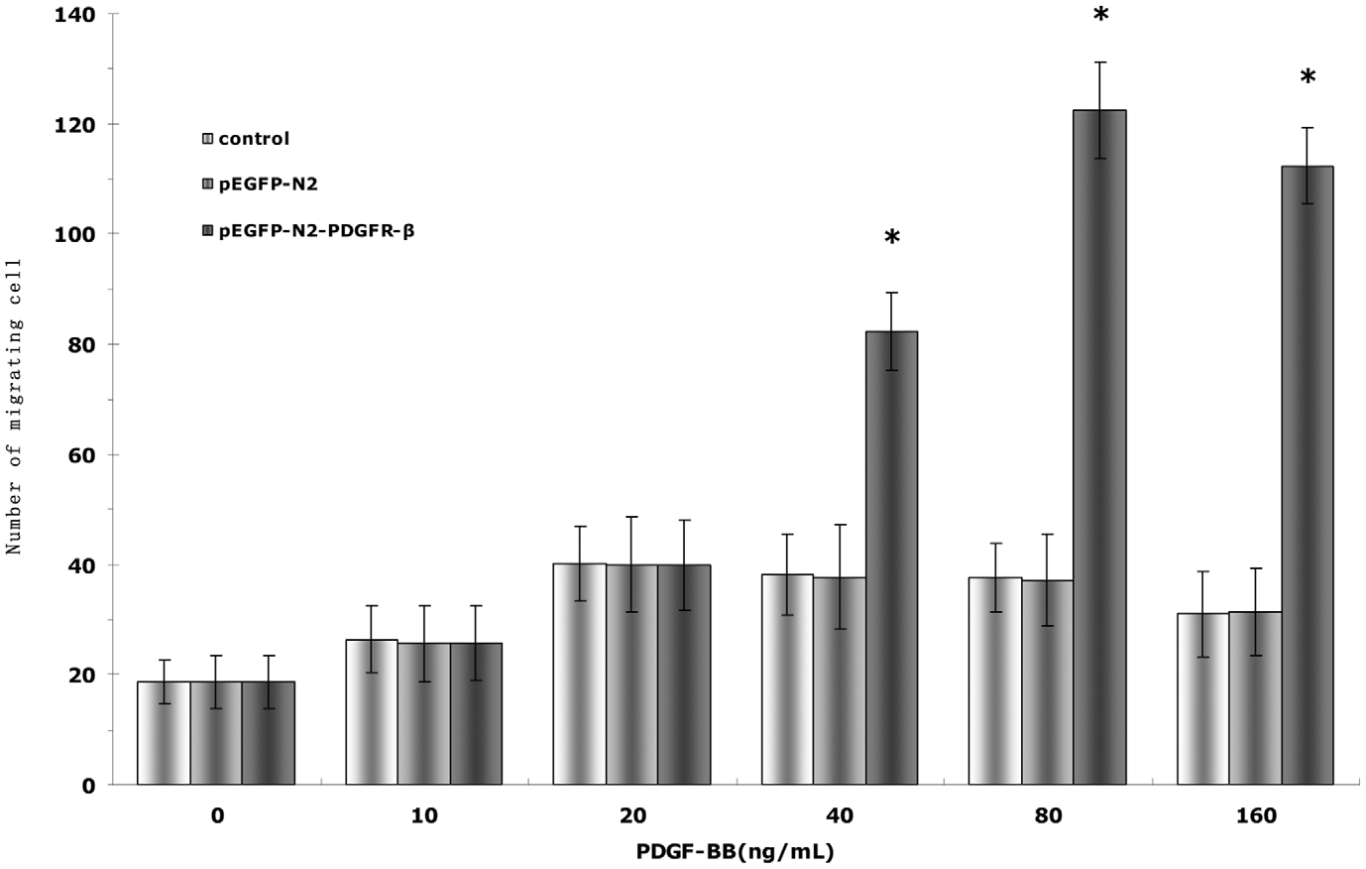
Effects of PDGFR-β overexpression on PDGF-BB-induced EPC migration. EPCs (untransfected,or transfected with either pEGFP-N2 or pEGFP-N2-PDGFR-β) were incubated with PDGF-BB of different concentrations. EPC migration was examined by the Transwell system. * *P*<0.01 vs. pEGFP-N2-PDGFR-β cells under 0 ng/mL PDGF-BB stimulation.

We further used the Transwell system to examine the effects of PDGFR-β over-expression on PDGF-BB-induced EPC migration. The main effects of concentration (F = 605.098, P<0.01) and group (F = 1422.843, P<0.01), as well as their interaction (F = 327.163, P<0.01) were all significant. The maximum migration induced by recombinant PDGF-BB occurred at 20 ng/mL in both the control and pEGFP-N2 groups and at 80 ng/mL in the pEGFP-N2-PDGFR-β group (Fig. 6). Since no difference was observed between the control and pEGFP-N2 groups, in subsequent migration experiments, only the control group and the pEGFP-N2-PDGFR-β group were compared, and the maximum migration concentration indentified were used to stimulate migration for the two groups. No significant difference in PDGF-BB-induced EPC migration was observed following increase in PDGF-BB concentration in the control and pEGFP-N2 groups (P>0.05) (Fig. 6). In contrast, EPC migration increased significantly with increase in PDGF-BB concentration (P<0.05), indicating that PDGF-BB-induced EPC migration was also enhanced by PDGFR-β over-expression.

### Effects of PDGFR-β over-expression on PDGF-BB-induced EPC tube-formation

**Figure 7:**
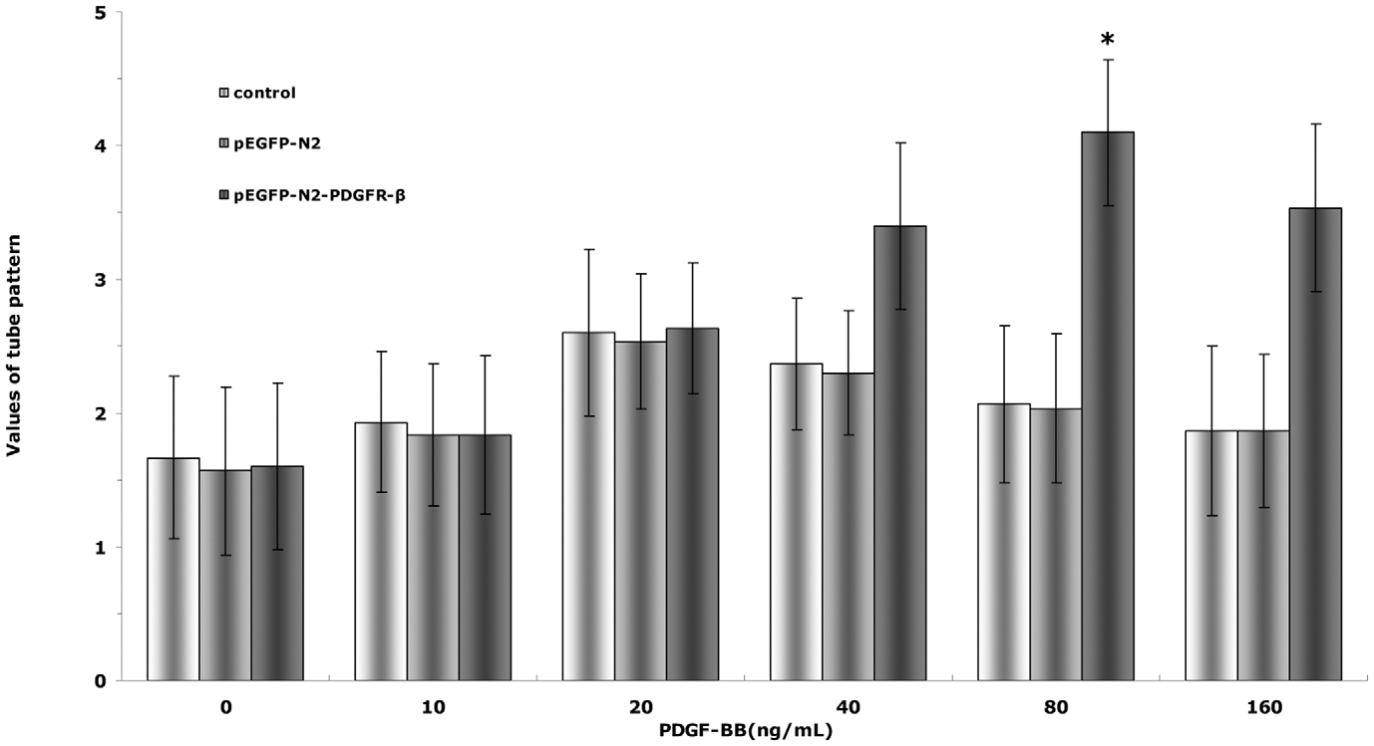
Effects of PDGFR-β overexpression on PDGF-BB-induced EPC tube formation. EPCs (untransfected,or transfected with either pEGFP-N2 or pEGFP-N2-PDGFR-β) were incubated with PDGF-BB of different concentrations. EPC tube formation was examined by the In Vitro Angiogenesis Assay Kit. * *P*<0.01 vs. pEGFP-N2-PDGFR-β cells under 0 ng/mL PDGF-BB stimulation.

We then used the In Vitro Angiogenesis Assay Kit to examine the effects of PDGFR-β over-expression on the tube formation of EPC. The main effects of concentration (F = 60.861, P<0.01) and group (F = 117.979, P<0.01), as well as their interaction (F = 26.599, P<0.01), were all significant. The maximum tube formation induced by recombinant PDGF-BB occurred at 20 ng/mL for both the control and pEGFP-N2 groups, and at 80 ng/mL for the pEGFP-N2-PDGFR-β group (Fig. 7). Since no difference was observed between the control and pEGFP-N2 groups, in the subsequent tube-formation experiments, only the control group and the pEGFP-N2-PDGFR-β group were compared, and the maximum tube-formation concentration indentified here were used for the two groups to stimulate tube-formation, respectively. There were no significant changes in PDGF-BB-induced EPC tube-formation with increase in PDGF-BB concentration in the control and pEGFP-N2 groups (P>0.05) (Fig. 7). In contrast, EPC tube-formation increased significantly with increase in PDGF-BB concentration(P<0.05), indicating that PDGF-BB-induced EPC tube-formation is also enhanced by PDGFR-β over-expression.

### The role of PI3K/Akt pathway in PDGF-BB-induced proliferation, migration, and tube-formation of EPCs

PDGFR-β and the activation of PI3K/Akt are both important for PDGF-BB to function [18], [28], [29]. We thus further tested whether the PI3K/Akt signaling pathway plays a role in the PDGF-BB-induced proliferation, migration, and angiogenesis of EPCs and whether this role is also important in the promotive effects of PDGFR-β over-expression on these PDGF-BB-induced phenotypes. AG1295 (a selective inhibitor of PDGFR), LY294002 (a PI3K inhibitor), and sc-221226 (an Akt inhibitor) were used to inhibit the activation of PDGFR or the PI3K/Akt signaling pathway.

**Figure 8:**
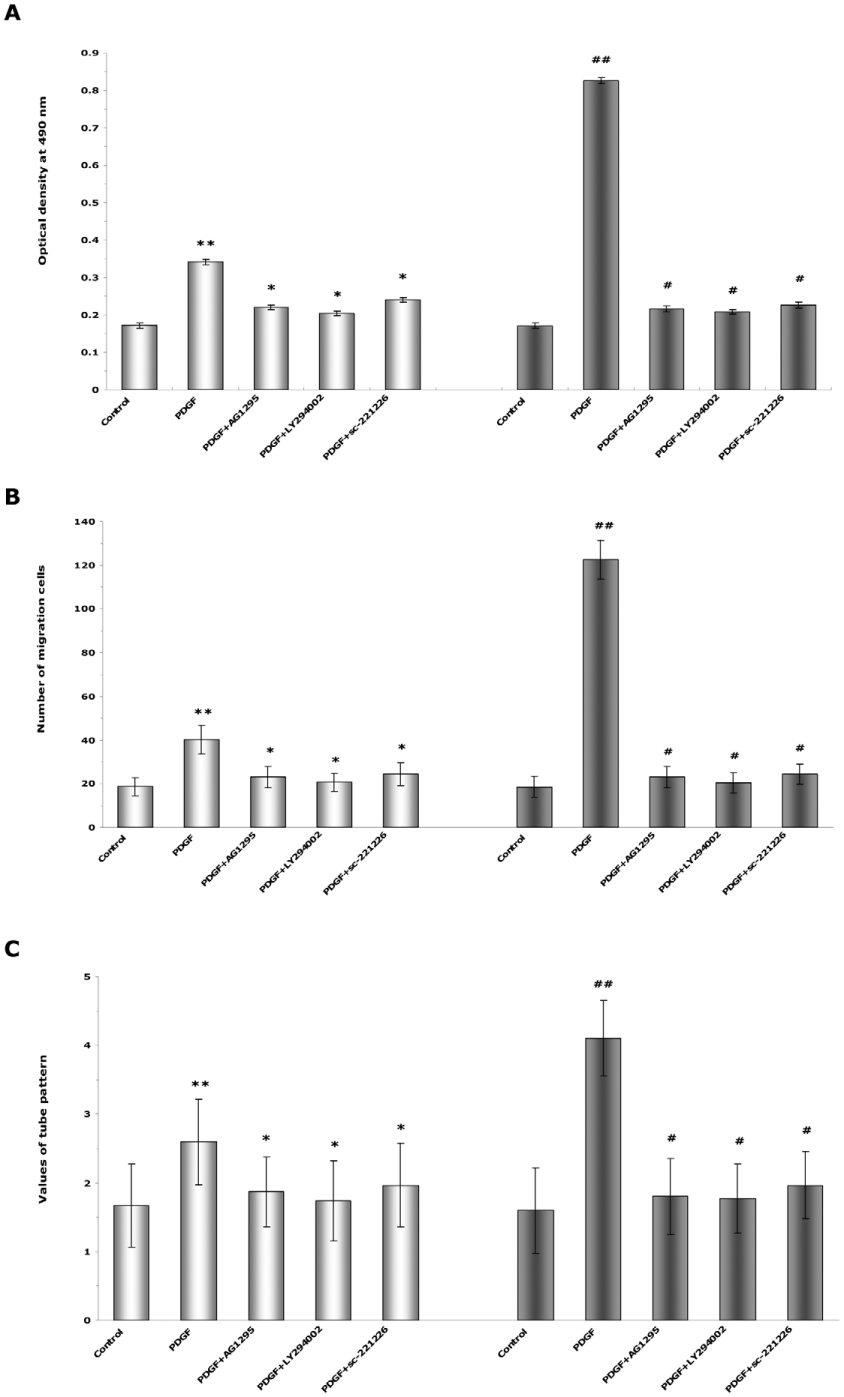
The role of PI3K/Akt pathway in PDGF-BB-induced proliferation, migration, and tube formation of EPCs. (**A, B, C**) Cells in the control group (bright bars, left half) and the pEGFP-N2-PDGFR-β group (dark bars, right half) were without pretreatment or pretreated with AG1295, LY294002, or sc-221226 for 1 h and then treated with 20 ng/mL PDGF-BB (for the control cells) or 80 ng/mL PDGF-BB (for the pEGFP-N2-PDGFR-β cells). PDGF-BB-induced proliferation (**A**), migration (**B**), and angiogenesis (**C**) of EPCs were significantly inhibited by pretreatment with AG1295, LY294002, or sc-221226. ** P<0.01 vs. control cells under 0 ng/mL PDGF-BB stimulation; * P<0.01 vs. control cells under 20 ng/mL PDGF-BB stimulation. ## P<0.01 vs. pEGFP-N2-PDGFR-β cells under 0 ng/mL PDGF-BB stimulation; # P<0.01 vs. pEGFP-N2-PDGFR-β cells under 80 ng/mL PDGF-BB stimulation.

**Figure 9:**
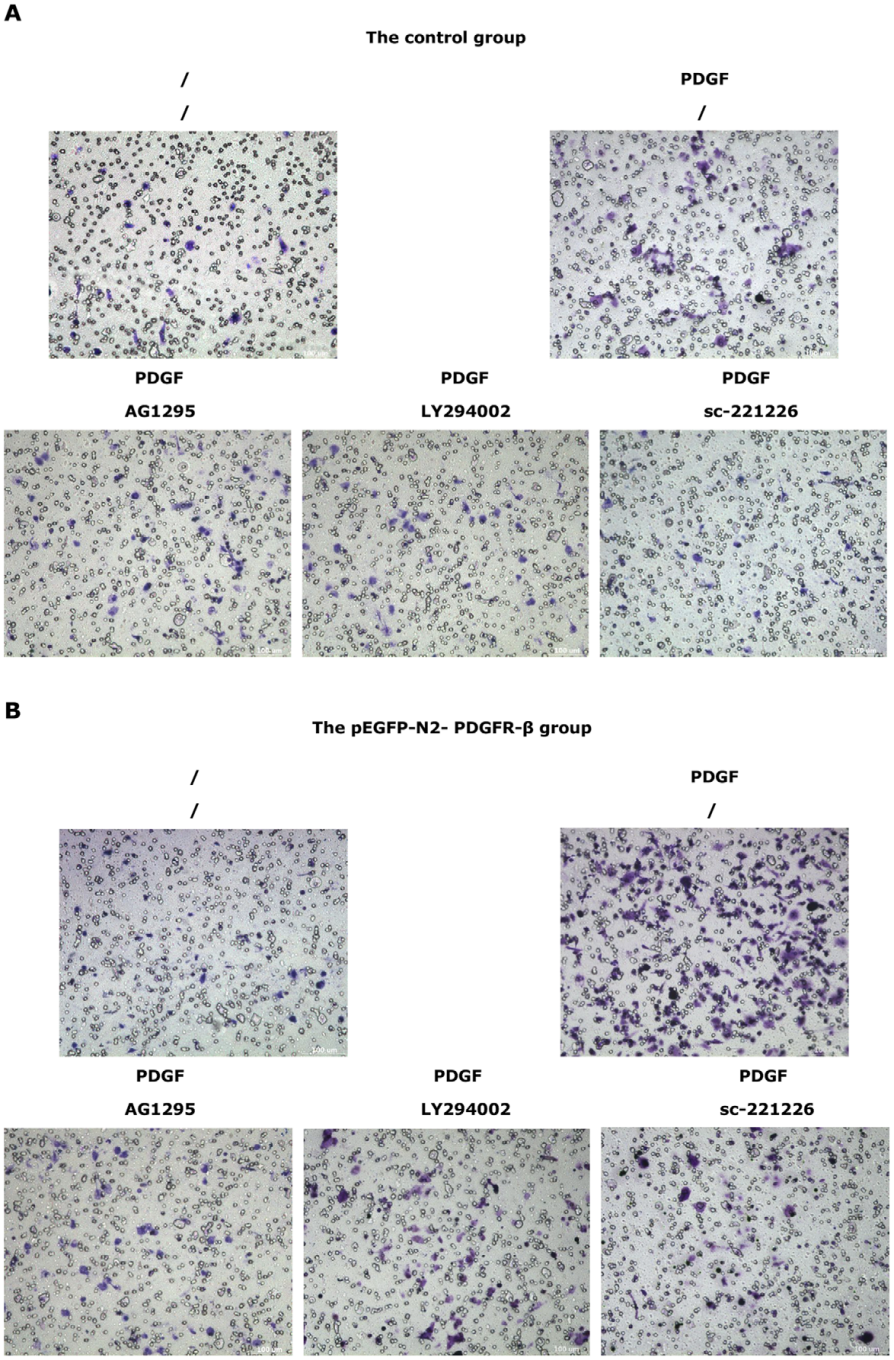
Representative photos of PDGF-BB-induced EPC migration by PI3K/Akt pathway. Cells in the control group (**A**) and the pEGFP-N2-PDGFR-β group (**B**) were without pretreatment or pretreated with AG1295, LY294002, or sc-221226 for 1 h and then treated with 20 ng/mL PDGF-BB (for the control cells) or 80 ng/mL PDGF-BB (for the pEGFP-N2-PDGFR-β cells). Scale bar = 100 µm.

**Figure 10:**
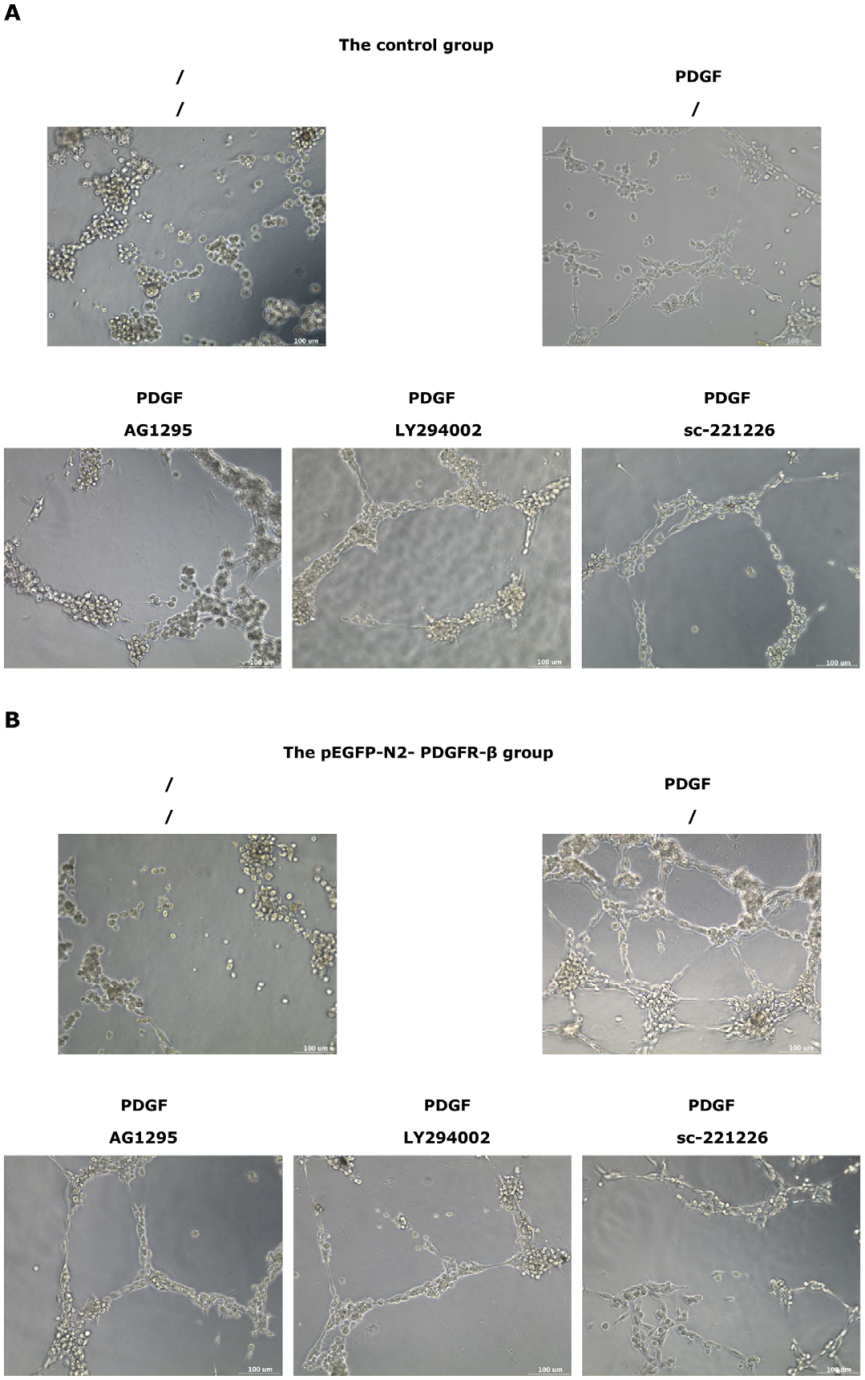
Representative photos of PDGF-BB-induced EPC angiogenesis in vitro by PI3K/Akt pathway. Cells in the control group (**A**) and the pEGFP-N2-PDGFR-β group (**B**) were without pretreatment or pretreated with AG1295, LY294002, or sc-221226 for 1 h and then treated with 20 ng/mL PDGF-BB (for the control cells) or 80 ng/mL PDGF-BB (for the pEGFP-N2-PDGFR-β cells). Scale bar = 100 µm.

Interestingly, the PDGF-BB-induced proliferation (Fig. 8A), migration (Fig. 8B), and angiogenesis (Fig. 8C) in the control group (by 20 ng/mL PDGF-BB) and the pEGFP-N2-PDGFR-β group (by 80 ng/mL PDGF-BB) were all significantly inhibited by the pretreatment of AG1295, LY294002, or sc-221226. These results indicated that the PI3K/Akt signaling pathway participated in the PDGF-BB-induced proliferation, migration (Fig. 9), and angiogenesis (Fig. 10) of EPCs.

In addition, the PI3K/Akt signaling pathway was required for PDGFR-β over-expression to enhance these PDGF-BB-induced phenotypes.

### Various inhibitors attenuate PDGF-BB-stimulated phosphorylation of PDGFR-β, PI3K, and Akt

**Figure 11:**
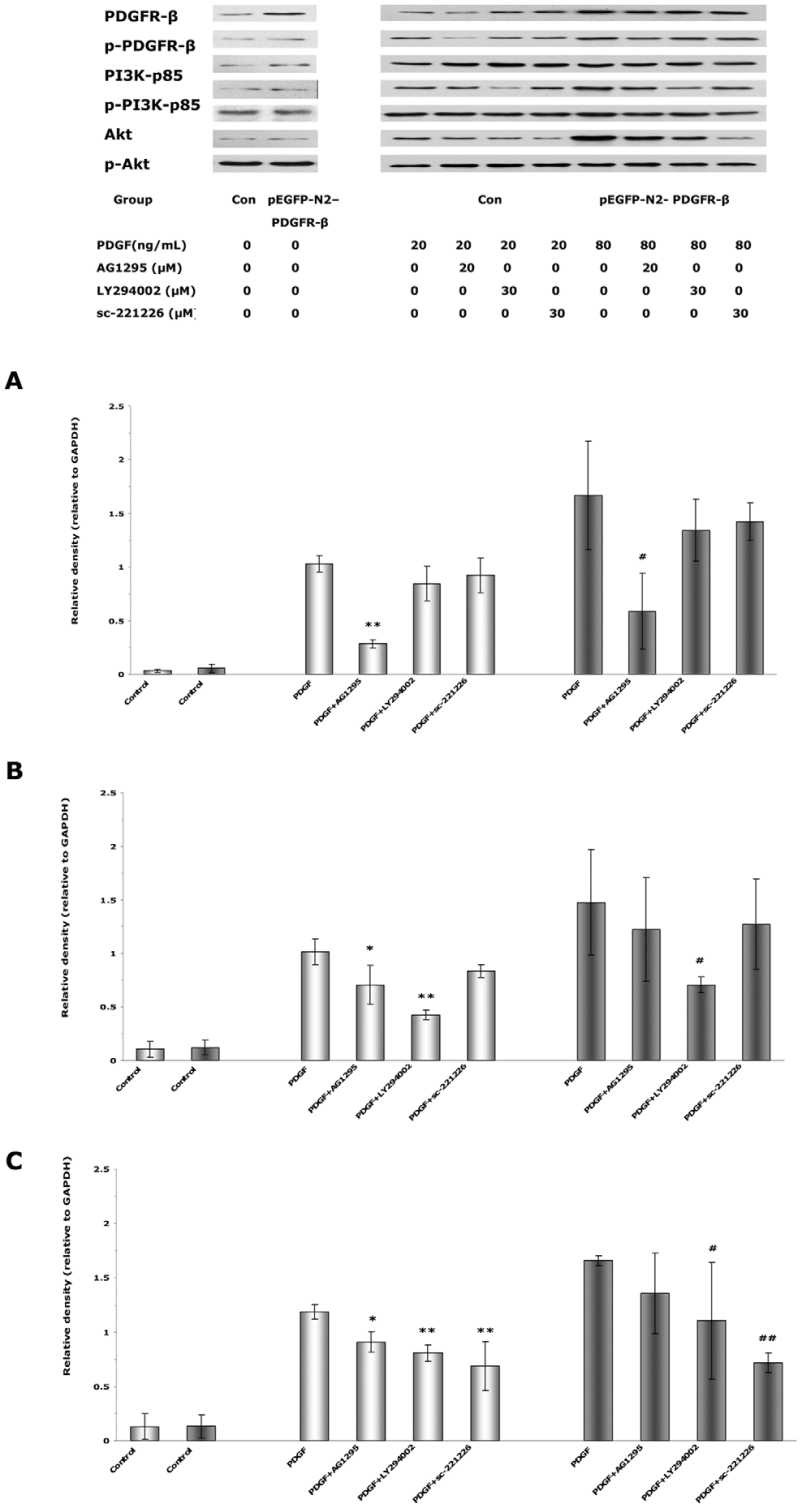
Pretreatment with various inhibitors attenuates PDGF-stimulated phosphorylation of PDGFR-β, PI3K, and Akt. (**A, B, C**) Cells in the control group (bright bars) and the pEGFP-N2-PDGFR-β group (dark bars) were pretreated with AG1295, LY294002, or sc-221226 for 1 h and then treated with 20 ng/mL PDGF-BB (for the control cells) or 80 ng/mL PDGF-BB (for the pEGFP-N2-PDGFR-β cells). PDGF-BB-induced phospho-PDGFR-β (**A**), phospho-PI3K (**B**), and phospho-Akt (**C**) were normalized to total PDGFR-β, PI3K, or Akt, respectively. * *P*<0.05 and ** *P*<0.01 vs. control cells under 20 ng/mL PDGF-BB stimulation. # *P*<0.05 and ## *P*<0.01 vs. pEGFP-N2-PDGFR-β cells under 80 ng/mL PDGF-BB stimulation.

The binding of PI3K to PDGFR-β has been shown to be important for the receptor mediated cell responses [30], [31]. To investigate whether the PDGFR-β/PI3K/Akt signaling pathway was involved in EPCs, the phosphorylation/activation levels of PDGFR-β, PI3K, and Akt were examined by western blot after exposure to PDGF-BB. Exogenous stimulation with PDGF-BB (20 ng/mL for the control cells and 80 ng/mL for the pEGFP-N2-PDGFR-β transfected cells) markedly upregulated the phosphotyrosine levels of PDGFR-β (Fig. 11A), PI3K (Fig. 11B), and Akt (Fig. 11C), confirming the activation of the PDGFR-β/PI3K/Akt signaling pathway.

The PDGFR inhibitor AG1295 (20 µM) significantly blocked the increase in p-PDGFR-β (Fig. 11A), p-PI3K (Fig. 11B), and p-Akt (Fig. 11C) in the control cells in response to PDGF-BB stimulation, indicating that PDGFR was critical for the PDGF-mediated activation of PI3K/Akt. In pEGFP-N2-PDGFR-β transfected cells, AG1295 also decreased the increase in p-PDGFR-β (Fig. 11A). However, the residual level of p-PDGFR-β under AG1295 treatment could still be high enough to stimulate the downstream PI3K/Akt signaling, generating p-PI3K (Fig. 11B) and p-Akt (Fig. 11C) at a level that was not significantly different from that in the absence of AG1295.

The PI3K inhibitor LY294002 (30 µM) significantly blocked the phosphorylation of PI3K (Fig. 11B) and Akt (Fig. 11C) in both control cells and pEGFP-N2-PDGFR-β transfected cells in response to PDGF-BB stimulation. In addition, the Akt inhibitor sc-221226 (30 µM) significantly blocked the phosphorylation of Akt (Fig. 11C) in both control cells and pEGFP-N2-PDGFR-β transfected cells in response to PDGF-BB stimulation.

## Discussion

EPCs are a special type of bone marrow-derived progenitor cells, showed EC features, and can differentiate into ECs [3]. EPCs have been indicated to play important roles in neovascularization and re-endothelialization. During postnatal neovascularization, blood vessels are regenerated from the proliferation and migration of not only ECs [3] but also EPCs [32], [33]. Infusion of *ex vivo* expanded EPCs has been shown to improve neovascularization in animal ischemia models [34]. In addition, EPCs play important roles in the regeneration of injured endothelium. The levels of EPCs can reflect vascular repair capacity and have been shown to be associated with endothelial function [8]. As EPCs contribute to neovascularization and reendothelialization, augmenting the number of EPCs and improving the functional activity of these cells may be an attractive therapeutic agents.

PDGF-BB has been well documented in contributing to vascular repair/remodeling in humans and animal models after vascular injury (recently reviewed in [35]). For instance, PDGF-BB expression is up-regulated in smooth muscle cells in rat carotid arteries after balloon injury to stimulate the proliferation and migration of small muscle cells [36]. On the other hand, inhibition of PDGF-BB signaling has been shown to reduce vascular repair/remodeling after angioplasty [37], [38], [39]. In the present study, we first confirmed the endogenous expression of PDGFR-β, and identified that the endogenous PDGFR-β was localized to the plasma membrane of spleen-derived EPCs. Furthermore, we found that the over-expression of PDGFR-β can promote the proliferation, migration, and angiogenesis of EPCs through enhancing the PDGF-BB bioavailability. Our results indicated that the transplantation of PDGFR-β over-expressed EPCs can be used as a novel therapy for the treatment of vascular injury. For instance, PDGFR-β transfected EPCs can be injected into the sites of vascular injury, where the PDGF-BB expression has been up-regulated. Based on our study, we propose that the exogenous PDGFR-β over-expression and the locally up-regulated PDGF-BB stimulation can take combined effects to amplify the activity of PDGF-BB stimulated EPCs and subsequently attenuate endothelial injury.

We note that PDGF and PDGFR also play roles in the autocrine stimulation of tumor growth as well as the paracrine stimulation of tumor-associated angiogenesis in a variety of human epithelial cancers (recently reviewed in [40]). For example, in glial tumors, the PDGF levels in tumor cells are correlated with the tumor grade [41]. The high expression level of PDGFR-β has been observed in ECs of gliomas [42] and breast cancers [43]. Consequently, anti-PDGFR-β therapies have been suggested in cancers where PDGFR-β-positive ECs are present [40].These observations indicated that the combined application of PDGFR-β and PDGF-BB might increase the risk of tumor progression. Hence, future work will be focused on the validation and optimization of this promising approach to optimize its efficacy in the treatment of vascular injury while maximally reducing the risk of tumor progression.

After tyrosine phosphorylation, PDGFR-β recruits PI3K, phospholipase Cγ1, Src family kinase, and phosphotyrosine phosphatase SHP-2 [44]. PI3K further activates Akt, which serves as a multifunctional regulator of cell survival, growth, and glucose metabolism [19], [45]. Since the simvastatin-induced phosphorylation of Akt in EPCs was first reported [20], several studies have shown that PI3K/Akt signaling is also required under other stimulations, including VEGF [23], estrogen [25], [26], and red wine [46], indicating that the PI3K/Akt pathway is important in EPC regulation. However, it remains unclear the role of PI3K/Akt signaling in EPC proliferation, migration, and angiogenesis stimulated by PDGF-BB and its receptor PDGFR-β.

In this study, we found that accompanying increased EPC activities, the phosphotyrosine levels of PDGFR-β, PI3K, and Akt all increased upon the stimulation of exogenous PDGF-BB. Investigation using the inhibitors for these three molecules (i.e. AG1295, LY294002, sc-221226) revealed that inhibiting the activity and phosphorylation of PDGFR-β, PI3K, and Akt could reduce PDGF-BB induced proliferation, migration, and angiogenesis of both the control EPCs and the PDGFR-β over-expressed EPCs. These observations indicated that the PI3K/Akt signaling pathway is crucial for the EPC responses induced by PDGF-BB and/or PDGFR-β. Our results are consistent with two recent reports in which bFGF, PDGF-BB, and PDGFR-β can promote the proliferation, migration and angiogenesis of EPCs [47], [48]. Importantly, in the present study, we demonstrated that the PI3K/Akt signaling pathway is important for PDGF-BB to affect EPCs. To our best knowledge, this is the first demonstration on the mechanisms underlying PDGF signaling mediated physiological changes of EPCs.

In conclusion, our study demonstrated that PDGFR-β, PI3K, and Akt are important participants following PDGF stimulation on EPCs. These molecules can promote re-endothelialization and postnatal neovascularization after vascular injury, and thus can be used as potential drug targets for the treatment of vascular injury.

## References

[1] Ross R (1999). Atherosclerosis—an inflammatory disease.. N Engl J Med.

[2] Behrendt D, Ganz P (2002). Endothelial function. From vascular biology to clinical applications.. Am J Cardiol.

[3] Asahara T, Murohara T, Sullivan A, Silver M, van der Zee R (1997). Isolation of putative progenitor endothelial cells for angiogenesis.. Science.

[4] He T, Smith LA, Harrington S, Nath KA, Caplice NM (2004). Transplantation of circulating endothelial progenitor cells restores endothelial function of denuded rabbit carotid arteries.. Stroke.

[5] Walter DH, Rittig K, Bahlmann FH, Kirchmair R, Silver M (2002). Statin therapy accelerates reendothelialization: a novel effect involving mobilization and incorporation of bone marrowderived endothelial progenitor cells.. Circulation.

[6] George J, Herz I, Goldstein E, Abashidze S, Deutch V (2003). Number and adhesive properties of circulating endothelial progenitor cells in patients with in-stent restenosis.. Arterioscler Thromb Vasc Biol.

[7] Mills NL, Tura O, Padfield GJ, Millar C, Lang NN (2009). Dissociation of phenotypic and functional endothelial progenitor cells in patients undergoing percutaneous coronary intervention.. Heart.

[8] Hill JM, Zalos G, Halcox JP, Schenke WH, Waclawiw MA (2003). Circulating endothelial progenitor cells, vascular function, and cardiovascular risk.. N Engl J Med.

[9] Werner N, Kosiol S, Schiegl T, Ahlers P, Walenta K (2005). Circulating endothelial progenitor cells and cardiovascular outcomes.. N Engl J Med.

[10] Nolan DJ, Ciarrocchi A, Mellick AS, Jaggi JS, Bambino K (2007). Bone marrow-derived endothelial progenitor cells are a major determinant of nascent tumor neovascularization.. Genes Dev.

[11] Ciarrocchi A, Jankovic V, Shaked Y, Nolan DJ, Mittal V (2007). Id1 restrains p21 expression to control endothelial progenitor cell formation.. PLoS One.

[12] Gao D, Nolan DJ, Mellick AS, Bambino K, McDonnell K (2008). Endothelial progenitor cells control the angiogenic switch in mouse lung metastasis.. Science.

[13] Hermanson M, Funa K, Hartman M, Claesson-Welsh L, Heldin CH (1992). Platelet-derived growth factor and its receptors in human glioma tissue: expression of messenger RNA and protein suggests the presence of autocrine and paracrine loops.. Cancer Res.

[14] Heldin CH, Westermark B (1999). Mechanism of action and in vivo role of platelet-derived growth factor.. Physiol Rev.

[15] Fredriksson L, Li H, Eriksson U (2004). The PDGF family: four gene products form five dimeric isoforms.. Cytokine Growth Factor Rev.

[16] Holmgren L, Glaser A, Pfeifer-Ohlsson S, Ohlsson R (1991). Angiogenesis during human extraembryonic development involves the spatiotemporal control of PDGF ligand and receptor gene expression.. Development.

[17] Hellström M, Kalén M, Lindahl P, Abramsson A, Betsholtz C (1999). Role of PDGF-B and PDGFR-beta in recruitmen of vascular smooth muscle cells and pericytes during embryonic blood vessels formation in the mouse.. Development.

[18] Heldin CH, Ostman A, Rönnstrand L (1998). Signal transduction via platelet-derived growth factor receptors.. Biochim Biophys Acta.

[19] Cantley LC (2002). The phosphoinositide 3-kinase pathway.. Science.

[20] Llevadot J, Murasawa S, Kureishi Y, Uchida S, Masuda H (2001). HMG-CoA reductase inhibitor mobilizes bone marrow-derived endothelial progenitor cells.. J Clin Invest.

[21] Urbich C, Knau A, Fichtlscherer S, Walter DH, Brühl T (2005). FOXO-dependent expression of the proapoptotic protein Bim: pivotal role for apoptosis signaling in endothelial progenitor cells.. FASEB.

[22] Urbich C, Dimmeler S (2005). Risk factors for coronary artery disease,circulating endothelial progenitor cells, and the role of HMG-CoA reductase inhibitors.. Kidney Int.

[23] Gerber HP, McMurtrey A, Kowalski J, Yan M, Keyt BA (1998). Vascular endothelial growth factor regulates endothelial cell survival through the phosphatidylinositol 3′-kinase/Akt signal transduction pathway. Requirement for Flk-1/KDR activation.. J Biol Chem.

[24] Bao H, Jacobs-Helber SM, Lawson AE, Penta K, Wickrema A (1999). Protein kinase B (c-Akt), phosphatidylinositol 3-kinase, and STAT5 are activated by erythropoietin (EPO) in HCD57 erythroid cells but are constitutively active in an EPO-independent, apoptosis-resistant subclone (HCD57-SREI cells).. Blood.

[25] Simoncini T, Hafezi-Moghadam A, Brazil DP, Ley K, Chin WW (2000). Interaction of oestrogen receptor with the regulatory subunit of phosphatidylinositol-3-OH kinase.. Nature.

[26] Zhao X, Huang L, Yin Y, Fang Y, Zhao J (2008). Estrogen induces endothelial progenitor cells proliferation and migration by estrogen receptors and PI3K-dependent pathways.. Microvasc Res.

[27] Yin Y, Huang L, Zhao X, Fang Y, Yu S (2007). AMD3100 mobilizes endothelial progenitor cells in mice,but inhibits its biological functions by blocking an autocrine/paracrine regulatory loop of stromal cell derived factor-1 in vitro.. J Cardiovasc Pharmacol.

[28] Zhou L, Takayama Y, Boucher P, Tallquist MD, Herz J (2009). LRP1 regulates architecture of the vascular wall by controlling PDGFRbeta-dependent phosphatidylinositol 3-kinase activation.. PLoS ONE.

[29] Choi KH, Kim JE, Song NR, Son JE, Hwang MK (2010). Phosphoinositide 3-kinase is a novel target of piceatannol for inhibiting PDGF-BB-induced proliferation and migration in human aortic smooth muscle cells.. Cardiovascular Res.

[30] Kundra V, Escobedo JA, Kazlauskas A, Kim HK, Rhee SG (1994). Regulation of chemotaxis by the platelet-derived growth factor receptor-beta.. Nature.

[31] DeMali KA, Whiteford CC, Ulug ET, Kazlauskas A (1997). Platelet-derived growth factor-dependent cellular transformation requires either phospholipase Cgamma or phosphatidylinositol 3 kinase.. J Biol Chem.

[32] Takahashi T, Kalka C, Masuda H, Chen D, Silver M (1999). Ischemia- and cytokine-induced mobilization of bone marrow-derived endothelial progenitor cells for neovascularization.. Nat Med.

[33] Reyes M, Dudek A, Jahagirdar B, Koodie L, Marker PH (2002). Origin of endothelial progenitors in human postnatal bone marrow.. J Clin Invest.

[34] Urbich C, Heeschen C, Aicher A, Dernbach E, Zeiher AM (2003). Relevance of monocytic features for neovascularization capacity of circulating endothelial progenitor cells.. Circulation.

[35] Siow RC, Churchman AT (2007). Adventitial growth factor signalling and vascular remodelling: potential of perivascular gene transfer from the outside-in.. Cardiovasc Res.

[36] Lindner V, Giachelli CM, Schwartz SM, Reidy MA (1995). A subpopulation of smooth muscle cells in injured rat arteries expresses platelet-derived growth factor-B chain mRNA.. Circ Res.

[37] Levitzki A (2005). PDGF receptor kinase inhibitors for the treatment of restenosis.. Cardiovasc Res.

[38] Ferns GA, Raines EW, Sprugel KH, Motani AS, Reidy MA (1991). Inhibition of neointimal smooth muscle accumulation after angioplasty by an antibody to PDGF.. Science.

[39] Deguchi J, Namba T, Hamada H, Nakaoka T, Abe J (1999). Targeting endogenous platelet-derived growth factor B-chain by adenovirus-mediated gene transfer potently inhibits in vivo smooth muscle proliferation after arterial injury.. Gene Ther.

[40] Raica M, Cimpean AM (2010). Platelet-derived growth factor (PDGF)/PDGF receptors (PDGFR) axis as target for antitumor and antiangiogenic therapy.. Pharmaceuticals.

[41] Majumdar K, Radotra BD, Vasishta RK, Pathak A (2009). Platelet-derived growth factor expression correlates with tumor grade and proliferative activity in human oligodendrogliomas.. Surg Neurol.

[42] Plate KH, Breier G, Farrell CL, Risau W (1992). Platelet-derived growth factor receptor-beta is induced during tumor development and upregulated during tumor progression in endothelial cells in human gliomas.. Lab Invest.

[43] Vrekoussis T, Stathopoulos EN, Kafousi M, Navrozoglou I, Zoras O (2007). Expression of endothelial PDGF receptors alpha and beta in breast cancer: Up-regulation of endothelial PDGF receptor beta.. Oncol Rep.

[44] Tallquist M, Kazlauskas A (2004). PDGF signaling in cells and mice.. CytokineGrowth Factor Rev.

[45] Datta SR, Brunet A, Greenberg ME (1999). Cellular survival: a play in three Akts.. Genes Dev.

[46] Huang PH, Chen YH, Tsai HY, Chen JS, Wu TC (2010). Intake of red wine increases the number and functional capacity of circulating endothelial progenitor cells by enhancing nitric oxide bioavailability.. Arterioscler Thromb Vasc Biol.

[47] Sufen G, Xianghong Y, Yongxia C, Qian P (2011). bFGF and PDGF-BB have a synergistic effect on the proliferation, migration and VEGF release of endothelial progenitor cells.. Cell Biol Int.

[48] Wyler von Ballmoos M, Yang Z, Völzmann J, Baumgartner I, Kalka C (2010). Endothelial Progenitor Cells Induce a Phenotype Shift in Differentiated Endothelial Cells towards PDGF/PDGFRb Axis-Mediated Angiogenesis.. PLoS ONE.

